# Estimation of Three-Dimensional Lower Limb Kinetics Data during Walking Using Machine Learning from a Single IMU Attached to the Sacrum

**DOI:** 10.3390/s20216277

**Published:** 2020-11-04

**Authors:** Myunghyun Lee, Sukyung Park

**Affiliations:** Department of Mechanical Engineering, Korea Advanced Institute of Science and Technology (KAIST), Daejeon 34141, Korea; m.hyunlee@kaist.ac.kr

**Keywords:** walking, biomechanics, machine learning, wearables, spring mechanics, ground reaction forces, center of pressure, joint torques, three dimensions

## Abstract

Kinetics data such as ground reaction forces (GRFs) are commonly used as indicators for rehabilitation and sports performance; however, they are difficult to measure with convenient wearable devices. Therefore, researchers have attempted to estimate accurately unmeasured kinetics data with artificial neural networks (ANNs). Because the inputs to an ANN affect its performance, they must be carefully selected. The GRF and center of pressure (CoP) have a mechanical relationship with the center of mass (CoM) in the three dimensions (3D). This biomechanical characteristic can be used to establish an appropriate input and structure of an ANN. In this study, an ANN for estimating gait kinetics with a single inertial measurement unit (IMU) was designed; the kinematics of the IMU placed on the sacrum as a proxy for the CoM kinematics were applied based on the 3D spring mechanics. The walking data from 17 participants walking at various speeds were used to train and validate the ANN. The estimated 3D GRF, CoP trajectory, and joint torques of the lower limbs were reasonably accurate, with normalized root-mean-square errors (NRMSEs) of 6.7% to 15.6%, 8.2% to 20.0%, and 11.4% to 24.1%, respectively. This result implies that the biomechanical characteristics can be used to estimate the complete three-dimensional gait data with an ANN model and a single IMU.

## 1. Introduction

Kinetic walking data (such as the ground reaction force (GRF) and joint torques) can be used as quantitative indicators for the diagnosis, rehabilitation, or sports performance [1,2,3,4]. The proportion of mediolateral (ML) components among the loads on the lower limb joints is approximately 30%, and the force or torque in the ML direction is used as an indicator to predict the injuries of athletes and for the initial diagnosis of patients [5,6,7]. Therefore, the gait dynamics in three dimensions (3D) (including the ML direction) must be analyzed. However, gait analysis usually requires a laboratory environment in which the kinetics and kinematics data can be measured with force plates and a motion capture system. For rehabilitation, visiting the laboratory or hospital causes temporal and spatial constraints, which are a considerable obstacle for patients.

Because the demand for diagnosis and gait analysis in daily life is increasing, the use of wearable monitoring devices that collect gait data is increasing [8]. However, these wearable devices (e.g., Galaxy Watch, Apple Watch, and Garmin) usually include a single inertial measurement unit (IMU), and only simple gait information (e.g., the step length, step frequency, and walking speed) can be provided. Accordingly, estimating meaningful dynamic information such as kinetics data is challenging. A significant problem of the current wearable devices is increasing the quality and quantity of the obtained data while maintaining the measurements simple.

Based on the limitations regarding the direct measurement of kinetics data with wearable devices, researchers have estimated kinetics data from kinematics data measured by wearable devices. For instance, Karatsidis et al. estimated accurately 3D GRFs from kinematics data of the entire body measured by IMU sensors with a distribution algorithm at various speeds [9]. However, wearing 17 IMUs on all segments of the body is infeasible in daily life. Moreover, an artificial neural network (ANN) was used to estimate unmeasured GRFs and reduce the number of IMUs. The 3D or vertical GRFs were estimated from the IMU measurements taken at the shanks or feet with an ANN [10,11]. In addition, a single IMU was attached to the sacrum to estimate the vertical GRFs [12].

There have been few attempts to estimate 3D joint torques with wearable devices compared to GRFs. In addition, only few researchers have used machine learning to predict 3D lower limb joint torques from walking data such as the lower limb joint angles, surface electromyography data, or GRFs [13,14]. In one study, the researchers estimated 3D joint torques with a wavelet neural network from eight surface electromyography and two GRF data points [13]. In another study, the joint torques were estimated from the kinematics data of the lower limbs, which were measured with a motion capture system with long short-term memory based on a recurrent ANN [14]. These researchers have estimated joint torques with data that must be measured in a laboratory environment. However, to use the joint torques with wearable devices, a measurement method with an IMU sensor must be proposed.

The estimation ability of an ANN is affected by the input variables [15,16,17]. To obtain efficient and accurate results, the suitable data type and size must be chosen [17]. Oh et al. used the self-organizing map technique (which selects the independent variables among the total variables) and a genetic algorithm (i.e., a general regression neural network, which selects highly correlated variables as the outputs of the selected variables) to predict the 3D GRFs during walking [16].

Recently, biomechanical characteristics were used to select efficient and appropriate input for the ANN [17]. Although human walking is an outcome of precise and complex control performed by numerous muscles, the relationships between the center of mass (CoM) and forces are reproduced by a simple mechanical system, such as a spring-loaded inverted pendulum (SLIP) model [18,19,20,21,22,23]. Based on confirmation that the lower limb segments are dependent on the motion of the CoM during walking [23], the kinetics and kinematics of the lower limbs could be approximated as the weighted sum of the CoM kinematics. The kinematics and kinetics of the lower limbs during walking were estimated with the IMU data attached to the sacrum based on the characteristics of the ANN, whose output is expressed as the weighted sum of the inputs [17]. However, because the spring mechanical relationship between the CoM and GRF is limited to two dimensions (2D), the previous study only estimated 2D walking data. Recently, it has been reported that a 3D spring walking model closely represents the relationship between CoM and GRF in 3D [24].

In this study, we proposed the CoM as a single IMU attachment location based on the biomechanical relationship between the CoM, GRF, and center of pressure (CoP) to estimate 3D kinetics data using an ANN. Data from 17 participants were measured by an IMU attached to the sacrum during walking on a treadmill at various walking speeds. We calculated the kinematics of the CoM by dividing the data into each stride and integrated the data twice. ANNs designed to estimate the GRF, CoP, and joint torques were trained and tested using the leave-one-subject-out (LOO) validation method.

## 2. Materials and Methods

**Figure 1:**
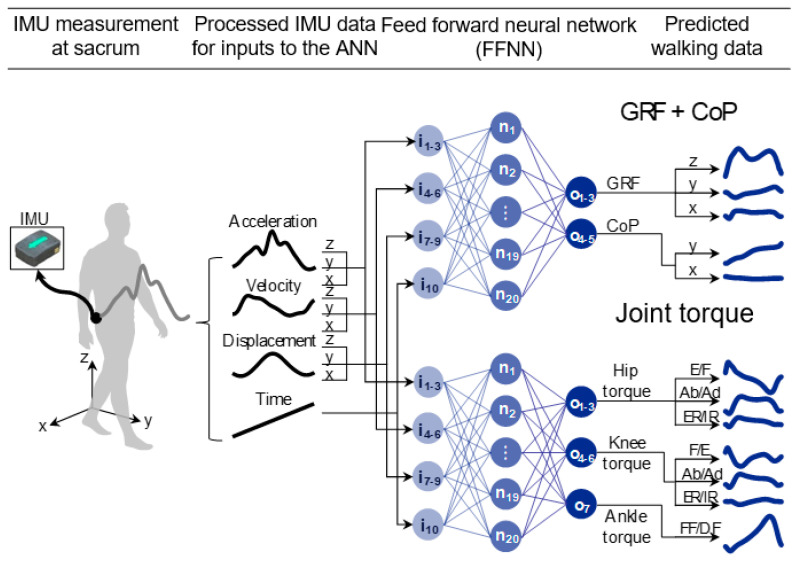
Schematics of the estimation procedures of lower limb kinetics data with a single inertial measurement unit (IMU) using an artificial neural network (ANN). One network predicted the ground reaction forces (GRFs) and center of pressure (CoP), and the other network predicted the joint torques. Measured and processed IMU data such as the acceleration, velocity, position, and time were fed into input nodes of the ANNs. Each ANN has 10 input nodes followed by one hidden layer of 20 nodes, and 5 or 7 output nodes, respectively. The output predictions are the vertical, anteroposterior (AP), and mediolateral (ML) GRFs, the AP and ML CoP, and the three-dimensional joint torques of hip, knee, and ankle.

We proposed a prediction method of the 3D kinetics data with a single IMU based on biomechanical characteristics, which is the spring mechanical relationship between the CoM, GRF, and CoP. An ANN with a single hidden layer was used to predict the 3D kinetics data due to the mechanical characteristics in which the GRF and CoP could be approximated by the weighted sum of the CoM kinematics during walking. We developed one model that predicted the GRF and CoP trajectories and another model that predicted the joint torques. Details of the experimental protocols, the data process from the IMU, and the estimation algorithm (Figure 1) are explained in later paragraphs.

### 2.1. Decision for a Single IMU Location Based on the 3D Compliant Model

We selected the optimal location for a single IMU that can predict the 3D kinetics data during walking by exploiting the mechanical relationship between the CoM, GRF, and CoP during walking in 3D [24]. Because an ANN model with a single hidden layer and a continuous sigmoidal function could approximate the continuous functions [25], we formulated the GRF and the CoP as a function of the CoM trajectory using the equations of motion of the CoM of the 3D SLIP model.

An approximated relationship between the CoM and the state variables of the 3D SLIP model was formulated (Equation (2)). The GRF and the trajectories of the CoP were formulated based on the state variables of the SLIP model (Equations (4) and (5)). Then, the GRF and the trajectories of the CoP were approximated as functions of the position of the CoM (Equation (6)). Based on these calculations, we could propose the CoM as the optimal location to estimate the 3D GRF and the CoP during walking. The details of the formulations follow.

**Figure 2:**
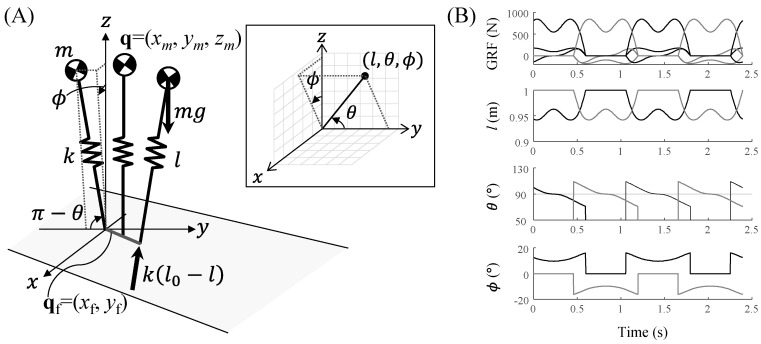
3D compliant walking model with a point foot control. (**A**) Model parameters and state parameters of the model and spherical coordinates with the *y*-axis as the zenith (in the inlet). The position of the CoM in 3D; *x_m_*, *y_m_*, and *z_m_* are the positions of the ML, AP, and vertical axes, respectively, and the model state variables *l*, *θ*, and *ϕ* are the spring length, the angle of the leg respect to the *y*-axis, and the angle of the projective leg to the *z*–*x* plane respect to the *z*-axis, respectively. (**B**) Example of the GRFs and the state variables during walking. The black and gray lines represent the variables for each leg, respectively. The *θ* s and *ϕ* s for walking solutions range from 68° to 112° and +/− 17°, respectively.

The position of the CoM of the 3D SLIP model, q = (*x_m_*, *y_m_*, *z_m_*), was represented as state variables, such as the leg length *l*, the angle with respect to the y-axis *θ*, the angle of the projective leg to the *z–x* plane with respect to the *z*-axis *ϕ*, the CoP excursion q_f_ = (*x*_f_, *y*_f_) during the stance phase, and subscript *hs* as the onset of the stance phase (Figure 2A), as follows:

(1) $$\left[\begin{array}{c} x_{m}\\ y_{m}\\ z_{m} \end{array}\right]=\left[\begin{array}{c} l\sin\theta \sin\phi +x_{f}+x_{f,hs}\\ l\cos\theta +y_{f}+y_{f,hs}\\ l\sin\theta \cos\phi \end{array}\right],$$

where *x*_f_ is sufficiently small and *y*_f_ is a sigmoidal function of time *t*.

By applying the small-angle approximation by substituting *θ*_⊥_+*π*/2 for *θ* (Figure 2B), we can obtain the augmented state *r* = [*l*, *θ*_⊥_, *ϕ*, *lθ*_⊥_, *lϕ*]^T^, which consists of the state variables of the 3D SLIP model, *l*, *θ*_⊥_, and *ϕ*, and their nonlinear term *lθ*_⊥_ and *lϕ*, expressed by the CoM position **q** and a sigmoidal function of time *t* as follows:

(2) $$r=W_{r}q_{t}+b_{r}$$

$$\mathrm{where}\;W_{r}=\left[\begin{array}{cccc} 0 & 0 & 1 & 0\\ 0 & -z_{m,hs}^{-1} & 0 & dz_{m,hs}^{-1}\\ z_{m,hs}^{-1} & 0 & 0 & 0\\ 0 & -1 & 0 & d\\ 1 & 0 & 0 & 0 \end{array}\right],\;q_{t}=\left[\begin{array}{c} x_{m}\\ y_{m}\\ z_{m}\\ f_{sigmoid}(t-a) \end{array}\right],\;b_{r}=\left[\begin{array}{c} 0\\ y_{f,hs}z_{m,hs}^{-1}\\ -\left(x_{f}+x_{f,hs}\right)z_{m,hs}^{-1}\\ y_{f,hs}\\ -\left(x_{f}+x_{f,hs}\right) \end{array}\right].$$

Based on the equation of motion of the 3D SLIP model (Equations (1) and (3)), the GRF **F** = [*F_x_*, *F_y_*, *F_z_*]^T^ and CoP can be expressed by the augmented state variable **r** as follows:

(3) $$\left[\begin{array}{c} F_{x}\\ F_{y}\\ F_{z} \end{array}\right]=\left[\begin{array}{c} k\left(l-l_{0}\right)\cos\theta_{⊥}\sin\phi \\ -k\left(l-l_{0}\right)\sin\theta_{⊥}\\ k\left(l-l_{0}\right)\cos\theta_{⊥}\cos\phi \end{array}\right],$$

(4) $$\begin{array}{c} F=W_{F}r+b_{F},\\ \mathrm{where}\;W_{F}=\left[\begin{array}{ccccc} 0 & 0 & -kl_{0} & 0 & k\\ 0 & kl_{0} & 0 & -k & 0\\ k & 0 & 0 & 0 & 0 \end{array}\right],\;b_{F}=\left[\begin{array}{c} 0\\ 0\\ -kl_{0} \end{array}\right] \end{array}$$

(5) $$\begin{array}{c} q_{f}=W_{f}q_{t}+W_{\mathrm{fF}}F+b_{f},\\ \mathrm{where}\;W_{f}=\left[\begin{array}{cccc} 1 & 0 & 0 & 0\\ 0 & 1 & 0 & 0 \end{array}\right],\;W_{\mathrm{fF}=}\left[\begin{array}{ccc} \frac{z_{hs}}{k\left(z_{hs}-1\right)} & 0 & 0\\ 0 & \frac{z_{hs}}{k\left(z_{hs}-1\right)} & 0 \end{array}\right],\;b_{f}=\left[\begin{array}{c} -x_{hs}\\ -y_{hs} \end{array}\right] \end{array}$$

(6) $$\left[\begin{array}{c} F\\ q_{f} \end{array}\right]=\left[\begin{array}{c} W_{F}W_{r}\\ W_{fF}W_{F}W_{r}+W_{f} \end{array}\right]q_{t}+\left[\begin{array}{c} W_{F}b_{r}+b_{F}\\ W_{fF}W_{F}b_{r}+W_{\mathrm{fF}}b_{F}+b_{f} \end{array}\right].$$

Because the GRF and CoP [**F**, **q_f_**]^T^ could be expressed as a weighted sum of the CoM position and time **q***_t_* and bias, we decided to use the CoM position and attach the IMU sensor to the sacrum most similar to the CoM in the body part to estimate the 3D kinetics data.

### 2.2. Experiments and Data Collection

Twenty young and healthy participants (12 females and 8 males, 24.7 ± 3.2 years) volunteered for the data collection, with average heights and mass of 166.7 ± 7.8 cm and 60.1 ± 8.9 kg. Data from three participants were removed due to equipment failure. Before the experiment, they signed informed consent forms approved by the KAIST Institutional Review Board on 15 October 2019 (KH2019–121). The participants walked on a customized split-belt treadmill at four different walking speeds (0.7, 1.0, 1.3, and 1.6 m/s) for 2 min at each speed.

The kinematics data of the CoM were measured by an IMU sensor (Trigno^TM^ Avanti sensors, Delsys, Natick, MA, USA) that was attached to the sacrum at a sampling frequency of 148 Hz. The GRFs and ground reaction moments were measured with two force plates (FP 6012^®^, Bertec, Columbus, OH, USA) underneath a split-belt treadmill at a sampling frequency of 200 Hz. Moreover, the trajectories of the optical markers attached to the body segments were measured by the motion capture system that included 10 optical cameras (Hawk^®^ and Osprey^®^, Motion Analysis Corporation, Rohnert Park, CA, USA) with sampling frequencies of 100 Hz. To use the steady-state walking data, 10 consecutive steps with the smallest deviation in the vertical GRF for each trial were used.

Subsequently, the kinetic and kinematics data were filtered with a fifth-order zero-phase Butterworth low-pass filter at cutoff frequencies of 20 and 10 Hz, respectively; all data were down-sampled to 100 Hz. The CoP trajectories were calculated with the GRFs and ground reaction moments. The 3D joint torques were calculated with the inverse dynamics of the 3D rigid body model, which consisted of six conical frustum-shaped segments for the lower limbs (e.g., the thighs, shanks, and feet) and a cylindrical segment for the trunk [26]. The segments were defined by optical markers attached to each lateral and medial joint. Furthermore, the 3D joint angles were determined based on the local frame axes of the segments, and the mass distribution was based on previous study [27].

### 2.3. Data Process of IMU Measurement

**Figure 3:**
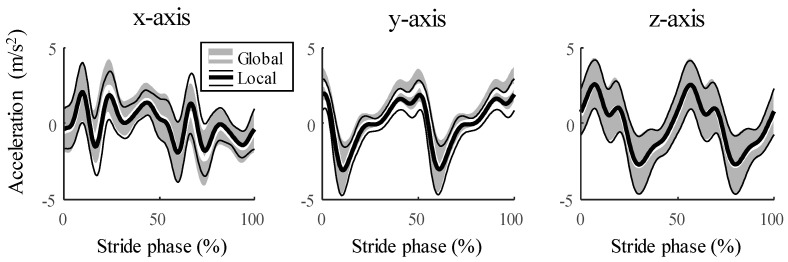
The average acceleration data from IMU during the stride phase for all walking speeds of all 17 participants. The averages and the standard deviations of the acceleration in the global frame are represented by black thick and thin solid lines, respectively, and the average and standard deviations of the acceleration in the local frame are represented by a white thick solid line and gray shade area, respectively. The percent of normalized root-mean-square errors (NRMSEs) of the acceleration in the local frame are 9.5 ± 3.3% in *x*-axis, 8.0 ± 3.5% in *y*-axis, and 2.3 ± 0.7% in *z*-axis.

We obtained the input data to the ANN model by calculating the velocity and the position of the sacrum by defining the gait events and estimating the walking speeds with the IMU data. We used the acceleration data in the local frame aligned with the forward direction, assuming that the difference between the acceleration in the local frame and that in the global frame is sufficiently small (Figure 3).

**Figure 4:**
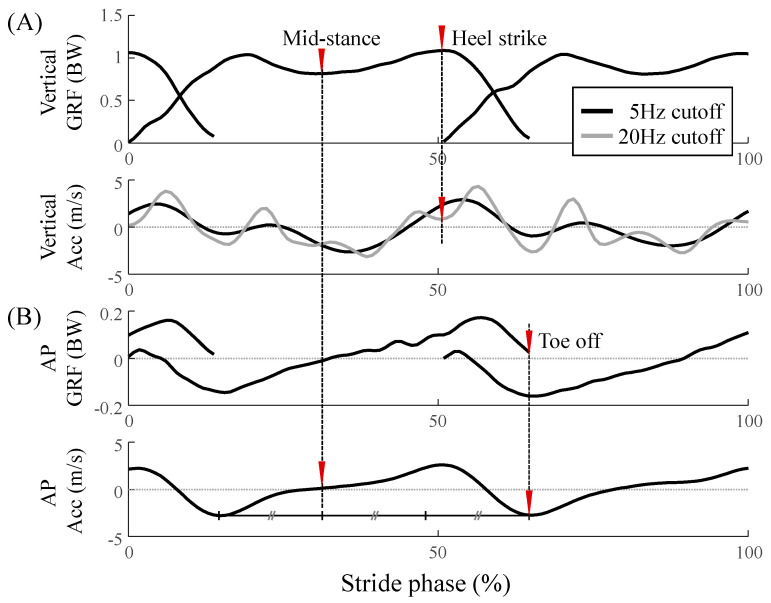
Schematics of the gait event detection algorithm. The CoM acceleration is compared to the GRFs in the (**A**) vertical and (**B**) AP directions to detect the heel strike (HS) and toe-off (TO), respectively. The mid-stance, HS, and TO are indicated by red inverted triangles. The data filtered by cutoff frequencies of 5 and 20 Hz are represented by the black and gray solid lines, respectively.

The acceleration data from the IMU sensor had to be segmented to be used for the stance phase. The gait events, the heel strike (HS) and toe-off (TO), were detected based on the sacral acceleration, by referring to the GRF (Figure 4) [17]. The cutoff frequencies of the low-pass filter were set heuristically to identify the acceleration characteristics for each event. Cutoff frequencies of 20 and 5 Hz were used for the HS and TO, respectively. First, to divide the step phase to include the bipedal support phase in the center, the mid-stance was used as the starting point; it re-occurred after one-third of the duration between the successive local minimum of the AP acceleration filtered at 5 Hz (Figure 4B). The HS was the last local minimum before the maximum of the step phase in the vertical acceleration filtered at 20 Hz, and the TO was the minimum of the step phase in the AP acceleration filtered at 5 Hz. When the detected HS came after the TO, the HS was identified as the previous local minimum of the originally detected HS until the HS was detected before the TO.

The next step was to calculate the velocity and displacement of the sacrum by integrating the acceleration data measured by the IMU sensor. The drift error that occurred during integration had to be removed, and the integral constant had to be determined. Both problems could be solved by assuming steady-state walking for each stride. Then, because the average velocity during a stride phase was zero, drift due to random errors could be linearly removed as follows:

(7) $$\hat{v}\left(t\right)=v\left(t\right)-\left(v\left(T\right)-v\left(0\right)\right)\cdot \frac{t}{T},$$

where $\hat{v}$, **v**, and *T* are the drift removal velocity, the velocity obtained by integrating the acceleration over time *t* during the stride phase, and the duration of the stride, respectively. Because the walking was steady-state, the integral constants were set such that the average velocities in the vertical and ML directions were zero.

However, because the average velocity in the anteroposterior (AP) direction was the walking speed, it was necessary to estimate the walking speed. Using the walking frequency and the average acceleration amplitude, which were correlated with the walking speed, the walking speed *v*_0_ was estimated as follows:

(8) $$v_{0}=\left(a_{1}f+a_{2}A+a_{3}\right)\sqrt{h\cdot g},$$

where *f* and *A* are the walking frequency and average magnitude of acceleration, respectively, and *a*_1_, *a*_2_, and *a*_3_ are the coefficient constants. The drift that occurred when calculating displacement by integrating the velocity could also be eliminated linearly by considering the average displacement for the stride phase. Although the average displacement in the vertical and ML directions was zero, the average displacement in the AP direction was determined by the walking speed. In contrast to the other direction, the data in the ML direction had the opposite sign depending on the leg side, so it was unified on one side. The leg side in the stance phase was determined by referring to the sign of the displacement in the 50% section of the stance phase and was unified with one leg. All kinematics data were processed by MATLAB 2017a.

### 2.4. Structure of ANN and Its Training and Test Procedures

In this study, the input and structure of the ANN model was designed based on the biomechanical relationship between the GRF, CoP, and CoM. Because the GRF and CoP were approximated based on the CoM position, the input to the ANN model was set according to the measured data of the IMU attached to the sacrum; in addition, the ANN model consisted of one hidden layer with a sigmoidal activation function [25]. Two ANN models were built: one for predicting the GRF and CoP and one for predicting the joint torques. The ANN models consisted of one input layer, one hidden layer, and one output layer. For both models, a 10 × 1 column vector was fed to 10 nodes of the input layer, which consisted of time *t* and the corresponding 3D kinematics data of the sacrum, which acted as a proxy of the CoM, such as (*t*, *x_m_*, *y_m_*, *z_m_*, *v_x_*, *v_y_*, *v_z_*, *a_x_*, *a_y_*, *a_z_*); *x_m_*, *y_m_*, and *z_m_* are the ML, AP, and vertical positions, and *v* and *a* are the velocity and acceleration of the sacrum measured at a specific time *t*, respectively. The hidden layer had 20 nodes with a sigmoidal function as the activation transfer function. Moreover, the two models had five and seven nodes for the output layer (consisting of 5 × 1 and 7 × 1 column vectors, respectively), which consisted of the 3D walking kinetics, such as (GRF_ML_, GRF_AP_, GRF_vertical_, CoP_ML_, CoP_AP_) and (*T*_Ab/Ad,hip_, *T*_E/F,hip_, *T*_ER/IR,hip_, *T*_Ab/Ad,knee_, *T*_F/E,knee_, *T*_ER/IR,knee_, *T*_PF/DF,ankle_); *T* is the joint torque, and the subscripts Ab, Ad, E, F, ER, IR, PF, and DF represent the abduction, adduction, extension, flexion, external rotation, internal rotation, plantar flexion, and dorsiflexion, respectively; in addition, the joint to which the torque was applied is indicated. The output layers used linear activation transfer functions.

All input data except the time were normalized according to the amplitudes of the data in the range 0 to 1. The output data, GRF, CoP, and joint torque were normalized by the body weight, foot length, and body mass, respectively. All input and output data for a stance phase were interpolated linearly to 201 points to match the length of the dataset to all walking speeds of all participants.

To validate the proposed estimation model, the datasets of a total of 17 participants were used to train and test the ANN with the LOO validation method. Each ANN model for each of the 17 participants was trained with the training set from the other 13 participants, and the datasets from the remaining three participants were used for validation stopping. Furthermore, the mean squared errors (MSEs) of the predicted and measured values were used as the loss function and Levenberg–Marquardt optimization for the backpropagation. When the MSE for the validation set failed to decrease or remained constant for 20 epochs, the training was stopped, and the parameters with the lowest MSEs for the validation set were chosen for the final models. To evaluate the estimation accuracy of the final models, all 40 stance phases from each participant and the normalized root-mean-square error (NRMSE) of the predicted values, which was normalized to the amplitude of the output of each stance phase, were used. The training of the neural network was conducted with the “train” function of MATLAB 2017a.

## 3. Results

The 3D kinetics data during walking were estimated with a single IMU attached to the sacrum, considered as the CoM using the ANN model. The predicted kinetics data are the vertical, AP, and ML GRF, the AP and ML CoP, the 3D joint torques at the hip and knee, and the ankle flexion torque.

**Figure 5:**
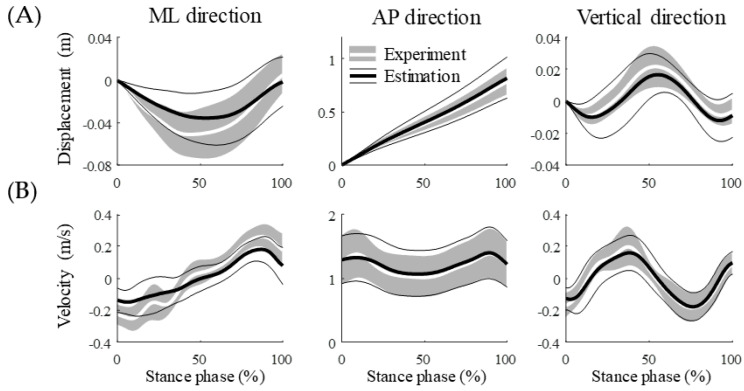
Average of the (**A**) displacement and (**B**) velocity of the sacrum during the stance phase for all walking speeds of all 17 participants. The averages and the standard deviations of the estimation results are represented by black thick and thin solid lines, respectively, and the average and standard deviations of the experimental data are represented by a white thick solid line and gray shade area, respectively.

We obtained walking data of the stance phase by estimating gait events such as the HS and TO using the acceleration from the IMU sensor, which were compared to the defined gait event by the GRF. The mean absolute error (MAE) of the HS and TO were 50 ± 41 and 13 ± 11 m/s, respectively, for a total of 680 stance phases in 17 participants (Table 1). Based on estimating gait events using the proposed method, the estimation accuracy for the TO was higher than that for the HS at all walking speeds. The estimation accuracy of HS did not correlate with speed (*r* = 0.045 and *p* = 0.24), whereas the accuracy of TO increased with increasing speed (*r* = −0.29 and *p* < 0.001). The MAE for the estimated walking speeds from all trials was 0.11 ± 0.11 m/s using the estimated gait events. We obtained the displacement and velocity of the sacrum for the stance phase by integrating acceleration from the IMU sensor (Figure 5). The estimated position and velocity of the sacrum had NRMSEs of 20% and 21%, respectively, compared with the kinematics data of an optical marker attached to the sacrum (Table 2).

**Figure 6:**
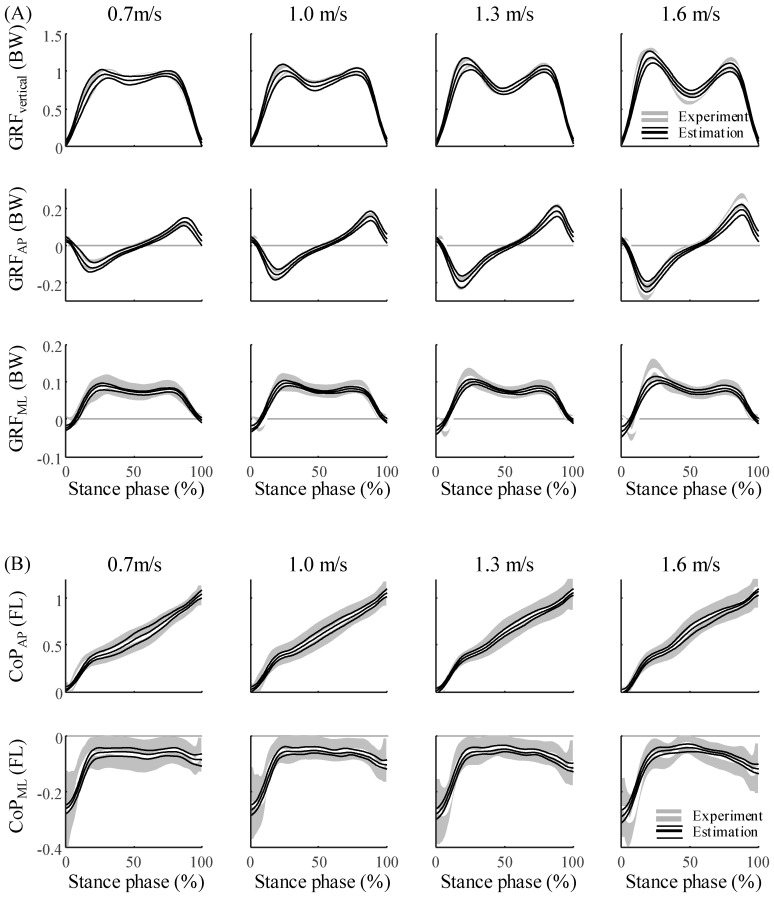
Estimation results and experimental data for (**A**) the GRF and (**B**) the CoP during the stance phase for all 17 participants. The averages and the standard deviations of the estimation results are represented by black thick solid lines and black thin solid lines, respectively, and the averages and standard deviations of the experimental data are represented by a white thick solid line and gray shaded area, respectively.

**Figure 7:**
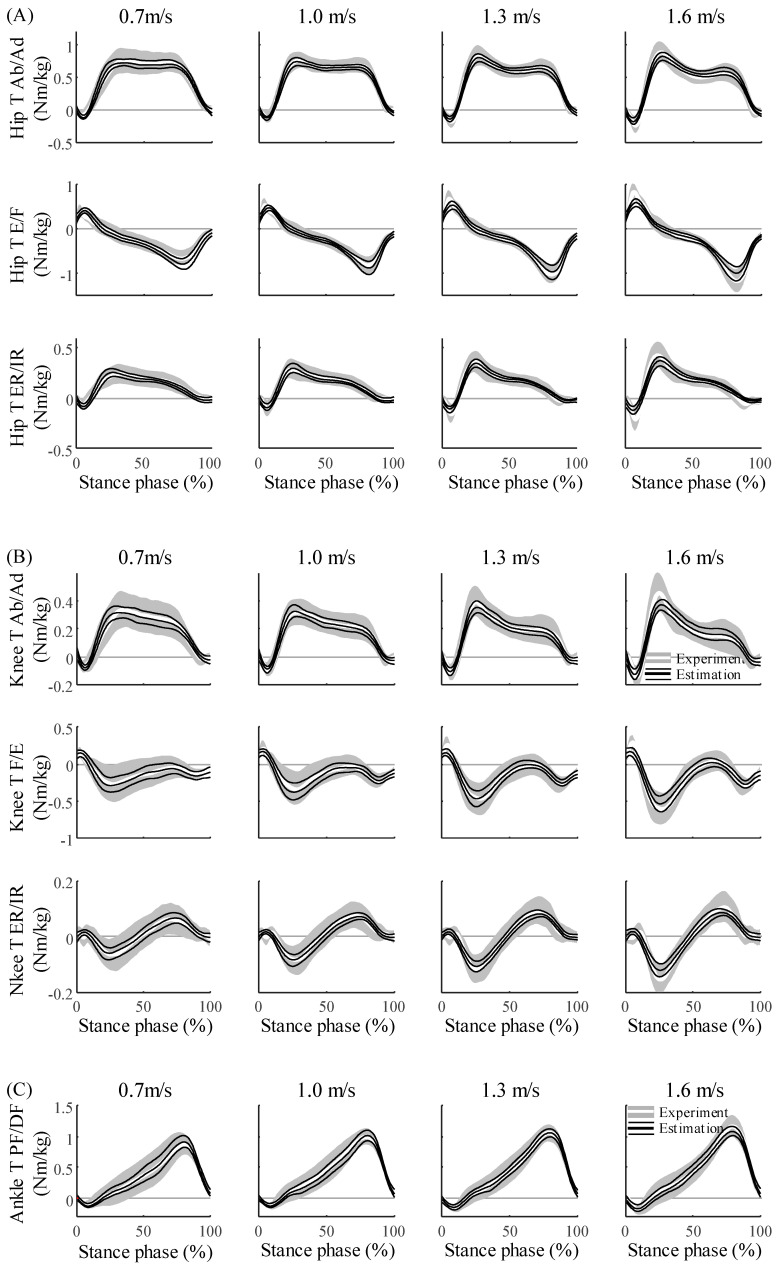
Estimation results and experimental data for the joint torque of (**A**) hip, (**B**) knee, and (**C**) ankle during the stance phase for all 17 participants. Ab/Ad, F/E (or E/F), ER/IR, and PF/DF represent abduction/adduction, flexion/extension (or extension/flexion), external rotation/internal rotation, and plantarflexion/dorsiflexion, respectively. The averages and the standard deviations of the estimation results are represented by black thick solid lines and black thin solid lines, respectively, and the averages and standard deviations of the experimental data are represented by a white thick solid line and gray shaded area, respectively.

The estimation of the 3D kinetics from the ANN agreed closely with the experimental data of all 17 participants (Figure 6 and Figure 7). The NRMSEs of the GRF, CoP, and joint torques were approximately 10%, 14%, and 17%, respectively (Table 3). The R^2^, the coefficient of determination of the GRF, CoP, and joint torques were approximately 0.81, 0.44, and 0.46, respectively. (Table 4). The GRF and joint torques estimated by the ANN showed the same trends across all walking speeds as the GRF measured with the force plate and joint torques that were calculated with the inverse dynamics. Except for the vertical GRF, all variables had the largest NRMSE at the slowest walking speed. The NRMSE was higher for joint torques than for the GRF and CoP (*p* < 0.001 for both), which could be approximated based on the weighted sum of the CoM.

## 4. Discussion

By designing the ANN model based on the 3D spring mechanics of walking, the 3D kinetics data during walking could be estimated with a single IMU sensor. The GRF and CoP were approximated by formulating them as functions of the CoM. The functional relationship between the CoM, GRF, and CoP enabled the use of an ANN with a single hidden layer for the estimation [25]. Based on this approximation, the ANN model, in which the sacrum kinematics were fed to the input nodes, could predict a total of 12 walking kinetics data points at various gait speeds with a single IMU sensor. The estimated GRF, CoP, and joint torques agreed closely with the measured kinetics data (Figure 6 and Figure 7). In previous studies, the 2D GRF and joint dynamics were only estimated with 2D sacrum acceleration based on the 2D spring walking model [17]. However, by proposing a 3D spring walking model that represents the 3D GRF and COP [24], the 3D kinetics data could be predicted with the 3D CoM kinematics. Furthermore, based on the training datasets from multiple participants, the applicability of the method to various participants despite their considerable variabilities was confirmed.

The performance of the ANN model proposed in this study reflects the walking reproducibility of the 3D SLIP model. Although the 3D SLIP model closely represents the GRF, the GRF reproducibility in the ML direction is lower than in the AP and vertical directions [24]. This may explain why the estimation accuracy in the ML direction is lower than those in the other directions. This low accuracy in the ML direction can be found in other existing 3D GRF estimation studies [9,16,28]. The high variability of the GRF in the ML direction compared to those of the other directions [29] would affect the low estimation accuracy. Because the ML CoP is much smaller than the AP CoP, the CoP moves only in the AP direction in the 3D SLIP model. The reproducibility of the model for the ML CoP is reflected in the performance of the ML CoP; thus, the error is larger than those of the GRF or AP CoP, and the SD of the estimation result is smaller than the SD of the experimental data. In addition, just as the GRF peak value of the 3D SLIP model did not reach the peak of the experimental data, the estimated GRF peak error was approximately 5% to 22% in this study, which exceeded the entire phase average error. The 3D joint torque had a large error compared to the GRF and COP. Because the 3D joint kinetics have no mechanical relationship to the CoM, the joint torques has a large estimation error compared to the GRF and CoP. Similar to those of the GRF peak, the joint torque peak errors were 16% to 27% (hip), 27% to 43% (knee), and 15% (ankle); thus, they were much greater than the entire phase average error. In the 2D case, the lower limb kinematics, lower limb kinetics, and GRF can be estimated based on the SLIP model with the ankle joint to reveal the dependency of the CoM and lower limb kinematics [17]. However, because the dependency of the CoM and lower limb kinematics is limited to the 2D case, the 3D kinematics could not be estimated with the ANN model and biomechanical characteristics of the CoM. In addition, by assuming a point mass, the rotation of the CoM and angular velocity of the IMU sensor were not considered despite the relationship between the joint torque and rotation. Insufficient input data for the estimation of the joint torque variability can decrease the estimated SD with respect to that of the experimental data. Therefore, using the angular velocity in the gyro as an additional input could lead to a similar SD with higher accuracy.

Considering the biomechanical characteristics while estimating the GRF and CoP can help to reduce drastically the necessary number of sensors. Several researchers have estimated the 3D GRFs and CoP with kinematics data and without considering the biomechanical characteristics during walking [9,16,28]. The 3D GRF and CoP could be predicted with the 3D accelerations and angular velocities from the 17 IMU sensors attached across the entire body. Despite the use of more sensors, the prediction results of the GRFs were similar to (for the AP and vertical directions) or only slightly more accurate (for the ML direction) than those of the proposed method; in addition, the prediction errors of the CoP were 1.5–2 times larger than those of the proposed method. These results imply a high correlation between the CoM kinematics and GRF and CoP. With machine learning, the 3D GRFs can be predicted based on the walking kinematics data without any biomechanical characteristics. The 3D GRF and joint torques were estimated with a general regression neural network and kinematics data recorded during walking. The motion data of the entire body were measured with optical markers in a laboratory environment; thus, their use in daily life was limited. The predictions in the AP and perpendicular directions exhibited smaller errors (approximately 1% to 2% lower); however, the ML directions exhibited errors that were more than 4% larger than the results of the proposed method. By using a feed-forward neural network with only two IMU sensors attached to both shanks [10], the accuracy of the GRF estimation was improved; this increases its potential for field applications. However, no LOO validation was used to validate the model; hence, the error may increase when the test data do not include data from the training participant. Furthermore, the requirement of multiple sensors remains. In this study, only a single IMU could estimate the 3D GRFs and CoP because biomechanical domain knowledge was used to design an ANN model.

Previously, the 3D lower limb joint torques were predicted with machine learning and gait data that must be measured in the laboratory [13,14]. Ardestani et al. estimated 3D joint torques with a wavelet neural network based on surface electromyography data and GRFs; the NRMSEs were 5% or less at all joint torques except during hip flexion (6.4%) [13]. In another study, Mundt et al. used the long short-term memory from the 3D joint angles of the lower limbs measured by a motion capture system to estimate the 3D joint torques [14]. Compared with the results of this study, only the NRMSE of the hip flexion had a larger error (18%); the NRMSEs of the other torques were smaller (6 to 15%). However, in this study, errors for only one participant were found; hence, the average NRMSE of all joint torques of all participants may increase. By using the biomechanical domain knowledge in the ANN design, the 3D joint torque can be estimated with a wearable device.

Estimating kinetics data with machine learning and biomechanical domain knowledge may resolve the tradeoff between the wearing convenience and data usability of wearable devices. The rehabilitation stage of a stroke patient can be assessed based on the propulsion of the paretic leg [30,31]. However, the patient must visit the motion analysis laboratory for the measurement. If a patient can obtain motion analysis data with comparable accuracy by using a wearable device without visiting the motion analysis laboratory, efficient rehabilitation diagnosis in daily life will be possible. Regarding the asymmetric walking of mildly hemiplegic patients, the difference in the AP GRF was approximately 50 N [30], and the minimal detectable changes were 2.9% and 4.7% body weight (BW) for the AP and vertical GRF when the stroke patients walk on a treadmill, respectively [32]. The proposed estimation method provided reliable estimations with average errors of 7.4%, 3.1%, and 2.2% (6.8%, 2.9%, and 2.1% for 0.7 m/s) BW for the vertical, AP, and ML GRFs, respectively; nevertheless, the error in the vertical direction of the proposed estimation method must be improved. The difference between the left and right knee adduction moments of osteoarthritis patients was 0.5% BW multiplied by height [33]. In this study, the knee adduction moment was estimated with an error of 0.06% BW multiplied by height. Considering the changes in the GRF and joint torque in stroke and arthritis patients, the proposed estimation method provides reliable prediction and diagnosis results. However, because the errors of the proposed method were average errors across the stance phase, the error can be larger or smaller for the clinically relevant parts, which could only be the part, such as at the highest or lowest value. Furthermore, using the method in rehabilitation and diagnosis of sports injuries requires further evaluation. In a previous study, the 3D GRF in the running and sidestep motions was estimated with a relative RMSE of 20% to 30% with a convolutional neural network (CNN) and data from an IMU attached to the sacrum [34]. The difference in the kinetics based on the change in the footstrike type during running is a GRF of 20% BW, knee adduction moment of 0.5 Nm/kg, and ankle plantar flexor moment of 0.6 Nm/kg [35]. Moreover, the risk of injury increased when the braking force exceeded 30% BW [36]. According to the error range, the proposed estimation method can be used for running.

Our study has several limitations in measuring and processing of the IMU data. In this study, data from walking on a treadmill was used to estimate gait kinetics. To apply the proposed method in daily life, it is essential to obtain a meaningful walking phase. Recently, a study has been reported in which repetitive walking phases were classified using IMU and GPS data that measured movements in daily life for ten days [37]. After the gait phase is determined, the step of gait event detection must be followed. If the gait event is estimated with higher accuracy, the CoM displacement and velocity can be more accurately calculated. Thus, the input to the ANN model can be more accurately provided to increase the model’s accuracy (not shown). In the previous study on 2D walking data prediction, the MAE of the HS estimation was 25 m/s, which was smaller than ours [17]. While the local minima for estimating HS was undoubtedly found for barefoot walking, it was sometimes not found in shoed walking, as in this study, so the estimation accuracy in HS was lowered. Wearing shoes reduces the effect of the GRF transmitted to the waist [38]. This is a similar reason why the accuracy of event detection is lower when using the IMU attached to the proximal segment away from the foot [39]. Therefore, using machine learning that can estimate the gait event with high accuracy, even with small signals at the waist, can improve the accuracy of estimating 3D kinetics data. Because the sacrum was used instead of the CoM, which has a relationship between GRF and spring dynamics, the input data to the ANN model has a difference with the velocity and position of the CoM [40,41]. The displacement magnitude of the sacrum in the vertical direction is larger than that of the CoM, which increases as walking speed increases [40]. Nevertheless, because the input is normalized to amplitude, the effect due to the amplitude difference between velocity and position is sufficiently small. The horizontal velocity of the CoM lags behind the sacrum; however, the difference decreases as walking speed increases [41]. The detected HSs were estimated to be ahead of the HSs defined by the force plates, in which case the error of the estimated velocity could be reduced. Nevertheless, the error of the velocity and displacement in other directions should be minimized through accurate detection.

The characteristics of the CoM dynamics were used as biomechanical knowledge to estimate the unmeasured gait data using ANN with only one IMU. By attaching the IMU to the sacrum, it was possible to estimate GRF, CoP, and lower limb kinetics during walking at various speeds—attributed the biomechanical properties that the GRF and CoP could be expressed as the CoM. The resulting reliable estimation accuracy suggests that the use of biomechanical characteristics and machine learning together can produce adequate results.

## Figures and Tables

**Table 1:** Mean absolute error (MAE) of the estimated gait events for all participants.

MAE (s)	0.7 m/s	1.0 m/s	1.3 m/s	1.6 m/s	Total
Heel strike	0.049 ± 0.037	0.051 ± 0.035	0.046 ± 0.035	0.056 ± 0.053	0.050 ± 0.041
Toe-off	0.018 ± 0.014	0.013 ± 0.009	0.010 ± 0.009	0.010 ± 0.006	0.013 ± 0.011

**Table 2:** Root-mean-square error (RMSE) and normalized RMSE of estimated displacement and velocity of the sacrum.

		Error	0.7 m/s	1.0 m/s	1.3 m/s	1.6 m/s	Total
Displacement	ML direction	RMSE (m)	0.021 ± 0.011	0.017 ± 0.009	0.014 ± 0.007	0.016 ± 0.009	0.017 ± 0.009
		NRMSE (%)	26.61 ± 13.27	27.45 ± 14.24	26.06 ± 13.17	27.67 ± 12.98	26.95 ± 13.41
	AP direction	RMSE (m)	0.05 ± 0.03	0.05 ± 0.03	0.05 ± 0.04	0.09 ± 0.08	0.06 ± 0.05
		NRMSE (%)	7.91 ± 5.09	6.30 ± 4.59	5.99 ± 5.18	9.40 ± 8.62	7.40 ± 6.23
	Vertical direction	RMSE (m)	0.005 ± 0.004	0.007 ± 0.004	0.010 ± 0.007	0.015 ± 0.015	0.009 ± 0.009
		NRMSE (%)	24.66 ± 17.13	24.41 ± 14.13	26.07 ± 16.22	28.60 ± 24.10	25.93 ± 18.32
Velocity	ML direction	RMSE (m/s)	0.08 ± 0.03	0.09 ± 0.03	0.09 ± 0.03	0.11 ± 0.04	0.09 ± 0.03
		NRMSE (%)	13.64 ± 6.19	15.98 ± 7.33	16.59 ± 5.90	17.61 ± 5.99	15.95 ± 6.53
	AP direction	RMSE (m/s)	0.11 ± 0.06	0.11 ± 0.07	0.13 ± 0.08	0.19 ± 0.14	0.14 ± 0.10
		NRMSE (%)	34.03 ± 17.68	29.54 ± 18.09	31.30 ± 23.68	39.29 ± 29.20	33.54 ± 22.90
	Vertical direction	RMSE (m/s)	0.04 ± 0.02	0.05 ± 0.02	0.06 ± 0.03	0.10 ± 0.08	0.06 ± 0.05
		NRMSE (%)	15.41 ± 7.01	14.17 ± 6.68	13.18 ± 6.62	14.57 ± 10.41	14.33 ± 7.86

**Table 3:** NRMSE of GRF, CoP, and joint torques (%) at each walking speed.

Walking Speed		0.7 m/s	1.0 m/s	1.3 m/s	1.6 m/s	Total
GRF	ML	17.75 ± 6.41	15.23± 5.50	14.19 ± 3.91	15.04 ± 2.78	15.55 ± 5.03
	AP	13.52 ± 4.38	8.31 ± 2.89	7.34 ± 2.46	7.67 ± 2.73	9.21 ± 4.06
	Vertical	6.86 ± 2.21	6.17 ± 1.81	6.08 ± 1.88	7.68 ± 3.15	6.70 ± 2.41
CoP	ML	21.12 ± 11.50	19.37 ± 9.66	18.62 ± 8.49	19.05 ± 8.24	19.54 ± 9.59
	AP	8.87 ± 6.15	8.36 ± 4.70	7.66 ± 4.37	8.00 ± 4.36	8.22 ± 4.96
Hip	Ab/Ad	15.38 ± 4.13	12.04 ± 4.17	11.50 ± 3.84	12.43 ± 4.20	12.84 ± 4.35
	E/F	16.08 ± 6.37	11.16 ± 4.64	9.29 ± 3.10	9.08 ± 2.44	11.40 ± 5.22
	ER/IR	23.66 ± 13.43	17.12 ± 8.45	14.47 ± 6.50	13.72 ± 5.85	17.24 ± 9.85
Knee	Ab/Ad	20.96 ± 9.40	16.88 ± 7.76	15.95 ± 10.49	16.33 ± 10.18	17.53 ± 9.70
	F/E	33.64 ± 17.11	25.77 ± 14.14	19.46 ± 9.12	17.47 ± 7.26	24.08 ± 14.01
	ER/IR	28.62 ± 12.59	22.69 ± 13.33	18.56 ± 8.19	16.16 ± 6.51	21.51 ± 11.54
Ankle	PF/DF	18.20 ± 9.57	12.80 ± 6.78	11.54 ± 5.65	11.78 ± 5.59	13.58 ± 7.57

**Table 4:** R^2^ values of GRF, CoP, and joint torques at each walking speed.

Walking Speed		0.7 m/s	1.0 m/s	1.3 m/s	1.6 m/s	Total
GRF	ML	0.54 ± 0.40	0.66 ± 0.25	0.70 ± 0.16	0.63 ± 0.14	0.63 ± 0.26
	AP	0.77 ± 0.15	0.91 ± 0.07	0.92 ± 0.05	0.91 ± 0.06	0.88 ± 0.11
	Vertical	0.94 ± 0.04	0.94 ± 0.03	0.94 ± 0.04	0.89 ± 0.09	0.93 ± 0.06
CoP	ML	−0.45 ± 1.98	0.02 ± 1.31	0.20 ± 1.18	0.27 ± 0.95	0.01 ± 1.43
	AP	0.82 ± 0.41	0.85 ± 0.37	0.90 ± 0.15	0.89 ± 0.17	0.86 ± 0.30
Hip	Ab/Ad	0.73 ± 0.16	0.83 ± 0.13	0.82 ± 0.13	0.77 ± 0.16	0.79 ± 0.15
	E/F	0.57 ± 0.39	0.79 ± 0.20	0.85 ± 0.11	0.87 ± 0.07	0.77 ± 0.26
	ER/IR	−0.03 ± 1.54	0.47 ± 0.75	0.60± 0.52	0.64 ± 0.38	0.42 ± 0.95
Knee	Ab/Ad	0.39 ± 0.79	0.56 ± 0.52	0.48 ± 1.15	0.41 ± 1.46	0.46 ± 1.04
	F/E	−1.17 ± 2.37	−0.31 ± 1.45	0.30 ± 0.68	0.51 ± 0.41	−0.17 ± 1.58
	ER/IR	−0.31 ± 1.58	0.04 ± 2.22	0.47 ± 0.69	0.61 ± 0.33	0.20 ± 1.45
Ankle	PF/DF	0.56 ± 0.52	0.80 ± 0.24	0.83 ± 0.19	0.82 ± 0.19	0.75 ± 0.33
